# Compound 19e, a Novel Glucokinase Activator, Protects against Cytokine-Induced Beta-Cell Apoptosis in INS-1 Cells

**DOI:** 10.3389/fphar.2017.00169

**Published:** 2017-03-29

**Authors:** Yoon Sin Oh, Eunhui Seo, Kaapjoo Park, Hee-Sook Jun

**Affiliations:** ^1^College of Medicine, Lee Gil Ya Cancer and Diabetes Institute, Gachon UniversityIncheon, South Korea; ^2^Gachon Medical Research Institute, Gil HospitalIncheon, South Korea; ^3^Department of Food and Nutrition, Eulji UniversitySeongnam, South Korea; ^4^Yuhan Research InstituteGyeonggi-do, South Korea; ^5^College of Pharmacy and Gachon Institute of Pharmaceutical Science, Gachon UniversityIncheon, South Korea

**Keywords:** compound 19e, glucokinase activator, beta-cell, apoptosis, cytokine, NAD-dependent protein deacetylase sirtuin -1

## Abstract

Previously, compound 19e, a novel heteroaryl-containing benzamide derivative, was identified as a potent glucokinase activator (GKA) and showed a glucose-lowering effect in diabetic mice. In this study, the anti-apoptotic actions of 19e were evaluated in INS-1 pancreatic beta-cells co-treated with TNF-α and IL-1β to induce cell death. Compound 19e protected INS-1 cells from cytokine-induced cell death, and the effect was similar to treatment with another GKA or exendin-4. Compound 19e reduced annexin-V stained cells and the expression of cleaved caspase-3 and poly (ADP-ribose) polymerase protein, as well as upregulated the expression of B-cell lymphoma-2 protein. Compound 19e inhibited apoptotic signaling via induction of the ATP content, and the effect was correlated with the downregulation of nuclear factor-κB p65 and inducible nitric oxide synthase. Further, 19e increased NAD-dependent protein deacetylase sirtuin-1 (SIRT1) deacetylase activity, and the anti-apoptotic effect of 19e was attenuated by SIRT1 inhibitor or SIRT1 siRNA treatment. Our results demonstrate that the novel GKA, 19e, prevents cytokine-induced beta-cell apoptosis via SIRT1 activation and has potential as a therapeutic drug for the preservation of pancreatic beta-cells.

## Introduction

Type 2 diabetes mellitus affected over 300 million people worldwide in 2013, and the prevalence of diabetes has increased noticeably over the past 50 years with a concomitant increase in the rates of obesity (Shaw et al., 2010). Diabetes is characterized by hyperglycemia, and insulin released by pancreatic beta-cells is the key hormone responsible for glucose homeostasis. In type 2 diabetes, beta-cells are damaged and become dysfunctional because of the persistently high levels of glucose, lipid, and inflammatory mediators released from the adipose tissues (Kahn et al., 2006). Thus, maintaining the pancreatic beta-cell mass may be a strategic approach for the prevention and treatment of diabetes.

Various anti-diabetic drugs targeting pancreatic beta-cells such as sulfonylureas, thiazolidinediones, incretin mimetics [glucagon-like peptide-1 (GLP-1) analogs] and G-protein coupled receptor 40 (GPR40) agonists have been developed (Stein et al., 2013). For example, rosiglitazone, a thiazolidinedione, protects against palmitate-induced cell death in beta-cell lines (Wu et al., 2013); exenatide, a GLP-1 receptor agonist, increases beta-cell proliferation and reduces beta-cell apoptosis *in vivo* (Vilsboll, 2009); and CNX-011-67, a GPR40 agonist, increases insulin secretion and reduces beta-cell apoptosis in the Zucker Diabetic Fatty rat, a diabetic animal model (Gowda et al., 2013).

Glucokinase, a member of the hexokinase family, is primarily expressed in hepatocytes, beta-cells, and hypothalamic neurons. Glucokinase facilitates the phosphorylation of glucose to glucose-6-phosphate, which is associated with a dual mechanism for lowering blood glucose concentrations by enhancing glucose uptake in the liver and increasing insulin secretion from pancreatic beta-cells (Matschinsky, 2009). Therefore, glucokinase has been an attractive target for anti-diabetic therapy over the past two decades. Several glucokinase activator (GKA) candidates have been shown to reduce blood glucose levels in diabetic animal models (Eiki et al., 2011; Gill et al., 2011; Park et al., 2013), including piragliatin, MK-0941, and AZD1656, which have advanced into clinical trials for patients with type 2 diabetes (Bonadonna et al., 2010; Meininger et al., 2011; Kiyosue et al., 2013; Wilding et al., 2013).

GKA has been shown to exert anti-diabetic effects by promoting proliferation and preventing apoptosis of beta-cells. Synthetic GKA compounds promote beta-cell proliferation by increasing the expression of insulin receptor substrate 2 (IRS-2) *in vivo* (Nakamura et al., 2012) and activating the IRS-2-AKT-Cyclin D2 pathway in INS-1 cells (Oh et al., 2014). Moreover, GKA shows anti-apoptotic effects against glucotoxicity-, oxidative stress- and endoplasmic reticulum (ER) stress-induced beta-cell death. These effects were probably through an increase in the glucokinase protein levels, phosphorylation of the apoptotic protein BCL2 associated agonist of cell death (BAD) and accelerated production of the reduced form of nicotinamide adenine dinucleotide and reduced form of nicotinamide adenine dinucleotide phosphate (Wei et al., 2009; Futamura et al., 2012; Shirakawa et al., 2013). Previously we reported that the anti-apoptotic effect of YH-GKA was the result of increase in interaction between glucokinase and mitochondrial membrane proteins (Oh et al., 2014). The physiological advantage of GKA-mediated signaling during glucotoxicity-induced beta-cell apoptosis has been investigated, but the effect of GKAs on cytokine-induced toxicity in beta-cells remains unknown.

As cytokines and nutrients trigger beta cell death via fundamentally different pathways (Cnop et al., 2005), the protective mechanisms of GKA might also be different depending on the type of toxic insult. Exposure of beta-cells to interleukin (IL)-1β combined with tumor necrosis factor (TNF)-α and/or interferon (IFN)γ causes cell death (Eizirik and Mandrup-Poulsen, 2001). IL-1β activates mitogen-activated protein kinase (MAPK) and the nuclear factor-κB (NF-κB) pathways, leading to the activation of inducible nitric oxide synthase (iNOS) and increase in nitric oxide (NO), which ultimately induces cell death. IFNγ induces apoptotic signals through a Janus kinase (JAK)–signal transducer and activator of transcription (STAT)-mediated signaling pathway, whereas TNF activates FAS-associated death domain protein (FADD) and MAPK pathways, which activate a series of caspase cysteine proteases (Vetere et al., 2014).

**FIGURE 1:**
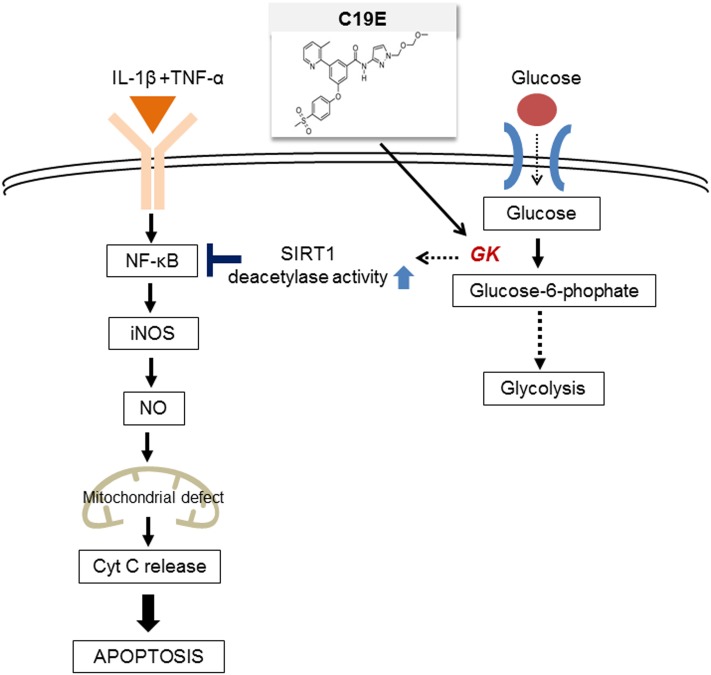
**The proposed molecular mechanisms of the compound 19e-mediated anti-apoptotic effect in INS-1 cells treated with cytokines**.

Novel synthetic GKAs, compound 19 and compound 19e (acetyoenyl- or heteroaryl- containing benzamide derivatives), were previously developed as active GKAs. Both compounds show glucose-lowering activities in C57BL/6J and *db/db* mice with no evidence for hypoglycemia risk (Park et al., 2014, 2015). The effect of these GKA compounds on beta-cell apoptosis was evaluated, and as only compound 19e showed anti-apoptotic effects against cytokine-induced beta-cell death, we investigated the mechanisms involved. We s found that compound 19e reduced cytokine-induced apoptotic signaling via inhibition of cytochrome c release. This was correlated with downregulation of NF-κB p65 and iNOS and was regulated by increased NAD-dependent protein deacetylase sirtuin-1 (SIRT1) deacetylase activity (**Figure 1**).

## Materials and Methods

### Materials

The novel GKAs, compound 19 and 19e, were prepared by Yuhan Research Institute (Yongin-si, South Korea) (Park et al., 2014) and were dissolved in dimethylsulfoxide. (2R)-2-(4-cyclopropanesulphonylphenyl)-N-(5-fluorothiazol-2-yl)-3-(tetrahydropyran-4-yl) propionamide (PSN-GK1), a GKA (Fyfe et al., 2007), and exendin-4 (Sigma-Aldrich, St. Louis, MO, USA), a GLP-1 receptor agonist, were used as positive controls. The following reagents were purchased from the indicated suppliers: annexin-V apoptosis detection kit (BD Transduction Laboratories, Palo Alto, CA, USA); antibodies against poly (ADP-ribose) polymerase (PARP) and cleaved PARP, cleaved caspase-3 and procaspase-3 and B-cell lymphoma 2 (Bcl-2) (Cell Signaling Technology, Beverly, MA, USA); and anti-NF-κB p65, cytochrome c, SIRT1, and secondary horseradish peroxidase-conjugated anti-mouse and anti-rabbit antibodies (Santa Cruz Biotechnology, Inc., Santa Cruz, CA, USA). All other biochemical reagents were from Invitrogen (Carlsbad, CA, USA), or Takara (Takara, Shiga, Japan).

### Cell Culture

The rat insulinoma cell line, INS-1 (passage 20 ∼ 30), was grown in Roswell Park Memorial Institute (RPMI)-1640 medium containing 11 mmol/l glucose. Fetal bovine serum (10%), 100 U/ml penicillin, and 100 μg/ml streptomycin were added to the culture medium. For experiments involving cytokine toxicity, INS-1 cells were incubated with 20 ng/ml TNF-α and 20 ng/ml IL-1β for 24 h. 0.025% of DMSO in media was used as a control. Cell viability was estimated using a cell counting kit that measures mitochondrial dehydrogenase activity (CCK-8) (Dojindo Laboratory, Kumamoto, Japan) as described previously (Oh et al., 2011).

### Annexin-V Staining

Early apoptosis was determined using an Annexin-V-phycoerythrin apoptosis detection kit, according to the manufacturer’s instructions. Briefly, the suspended and adherent cells were pooled, washed twice with ice-cold PBS, and resuspended in binding buffer. The cell suspension was incubated with annexin-V-phycoerythrin and 7-amino-actinomycin at room temperature. After incubation, stained cells were analyzed by flow cytometry (FACS Calibur, BD Transduction Laboratories) using CELLQuest software.

### Nuclear Protein Extract Preparation

The cells were homogenized in buffer containing 10 mM HEPES-KOH, pH 7.9, 10 mM KCl, 1.5 mM MgCl_2_, 1 mM dithiothreitol, 0.1% NP40, and protease inhibitors. Nuclei were separated by centrifugation (7200 × *g*) for 10 min at 4°C and resuspended in nuclei resuspension buffer (10 mM HEPES-KOH, pH 7.9, 400 mM KCl, 0.1 mM EDTA, 25% glycerol, and protease). The mixture was stirred gently for 10 min at 4°C and centrifuged at 1500 × *g*. The nuclear protein extract was recovered in the supernatant. Protein concentration was determined by using a BCA protein assay kit (Pierce, Waltham, MA, USA).

### Western Blot Analysis

INS-1 cells were extracted in lysis buffer (50 mM Tris-HCl, pH 7.4, 1% Triton X-100, 150 mM NaCl, 1 mM EDTA, 1 mM NaF, 1 mM Na_3_VO_4_, 1 mM phenylmethylsulfonyl fluoride, and 1 mM protease inhibitor cocktail). Thereafter, 35 ∼ 40 μg of protein from the lysates was resolved by 15% SDS-PAGE, transferred onto nitrocellulose membranes, and blocked with TBS containing Tween 20 in 3% non-fat dry milk. The membranes were incubated with specific antibodies and visualized by blotting with horseradish peroxidase-conjugated secondary antibodies. Signals were detected using the ECL detection system (Pierce, Rockford, IL, USA). The intensities of the bands were normalized to the actin band using Image J software (National Institute of Health, Bethesda, MD, USA).

### Quantitative Real-Time PCR

Isolation of total RNA was performed according to the manufacturer’s recommended protocol for Trizol. Extracted RNA was treated with DNase I and reverse transcribed to single-strand cDNA using oligo(dT) primer with PrimeScript^TM^ RTase (Takara). Quantitative real-time PCR (qRT-PCR) analysis was performed using SYBR mater mix (Applied Biosystems) using the ABI 7900 Real-time PCR system according to the protocols provided by the manufacturer (Applied Biosystems). The sequences of the primer pairs are as follows: iNOS (forward) 5′-CTCACTGTGGCTGTGGTCACCTA-3′ and (reverse) 5′-GGGTCTTCG GGCTTCAGGTTA-3′, SIRT1 (forward) 5′-GAGCAGGTTGCAGGAATCCA-3′ and (reverse) 5′-GCAAGATGCTGTTGCAAAGG-3′ and cyclophilin (forward) 5′-GGTCTTTGGGAAGGTGAAAGAA-3′ and (reverse) 5′-GGTCTTTGGGAAGGTGAA AGAA-3′. The relative mRNA transcript levels were calculated according to the 2^-Δ^*C*^T^ method, in which Δ*C*T represents the difference in threshold cycle values between the target mRNA and the cyclophilin internal control.

### Measurement of Cellular Adenosine Triphosphate

Intracellular adenosine triphosphate (ATP) content was measured using a CellTiter-Glo luminescence kit (Promega Life Science, Madison, WI, USA). The test was performed according to the manufacturer’s recommendations. Cells were mixed with the CellTiter-Glo reagent and lysed by vortexing. After incubating at room temperature for 10 min, luminescence was recorded by a microplate reader. The quantity of ATP was calculated using an ATP standard curve and the ATP content was normalized by the protein amount.

### Measurement of SIRT1 Deacetylase Activity

Nuclear protein was extracted and SIRT1 deacetylase activity was measured using a SIRT1 activity assay kit according to the manufacturer’s protocol (Abcam, Cambridge, MA, USA). Fluorescence intensity was measured for 30 min at 2 min intervals using a microtiter plate fluorometer with excitation at 340 nm and emission at 460 nm. SIRT1 deacetylase activity was normalized by the protein content.

### siRNA Transfection

The cells were transiently transfected with 100 nM of SIRT1 siRNA or scrambled siRNA (Santa Cruz Biotechnology, Santa Cruz, CA, USA) using Lipofectamine RNAi MAX (Invitrogen, Carlsbad, CA, USA) reagent in accordance with manufacturer’s protocol. After 24 h, the medium was replaced with cytokine mixtures with or without GKAs.

### Statistical Analysis

Data were expressed as the mean ± SEM and were analyzed using one-way, two-way, or repeated measurements analysis of variance (ANOVA). Differences between the treatment groups were analyzed by the Duncan’s multiple range test.

## Results

### Compound 19e Treatment Protects INS-1 Cells from Cytokine Mixture-Induced Cell Death

**FIGURE 2:**
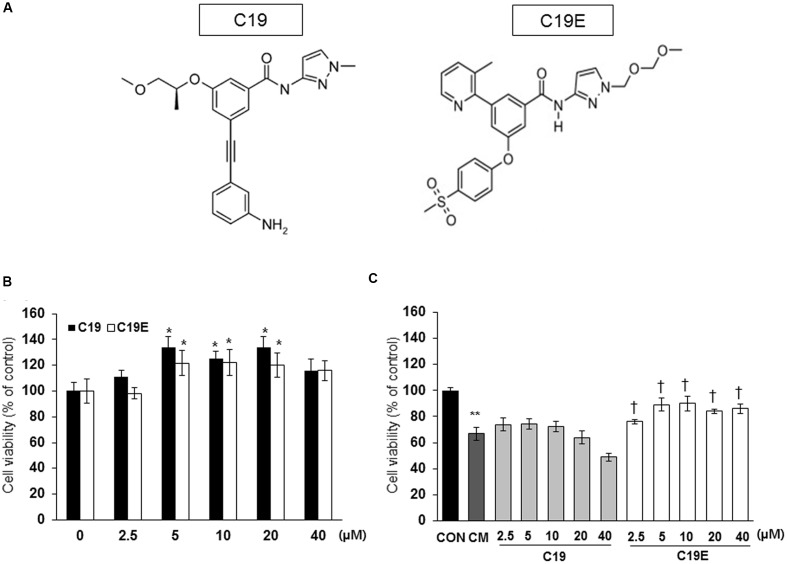
**Effect of GKA compounds on cell viability. (A)** Structure of compound 19 (C19) and compound 19e (C19E) **(B)** INS-1 cells were treated with various concentrations of C19 or C19E for 24 h, and viability was determined using a cell counting kit. **(C)** INS-1 cells were treated with 20 ng/ml of IL-1β and 20 ng/ml TNF-α (CM) in the absence or presence of various concentrations of C19 or C19E. After 24 h, cell viability was determined by using a cell counting kit. Results represent the mean ± SEM from triplicate experiments. ^∗^*P* < 0.05 vs. 0, ^∗∗^*P* < 0.05 vs. CON, ^†^*P* < 0.05 vs. CM alone.

**Figure 1** shows the proposed pathway for the action of compound 19e. To elucidate this pathway, we first examined whether synthesized GKA compounds (compound 19 and compound 19e) (**Figure 2A**), previously identified as potent GKAs (Park et al., 2014, 2015), exert toxicity on INS-1 cells. After 24 h of treatment, cytotoxicity was not observed at any concentration (2.5 ∼ 40 μM), and an increased cell viability was demonstrated with concentrations of 5, 10, and 20 μM of the compounds (**Figure 2B**). To investigate whether the compounds exert anti-apoptotic effects, INS-1 cells were incubated with a cytokine mixture, which is known to induce beta-cell death, in the absence or presence of compound 19 or 19e (2.5 ∼ 40 μM), and the cell viability was determined. After 24 h of treatment, the mixture of interleukin (IL)-1β and TNF-α (20 ng/ml of IL-1β and 20 ng/ml of TNF-α) caused a marked reduction of viable cells compared with the control cells, and compound 19e significantly reduced the toxic effect (**Figure 2C**). Compound 19 did not exert a protective effect at either low or high concentrations.

### Compound 19e Treatment Reduced Cytokine Mixture-Induced Beta-Cell Apoptosis

**FIGURE 3:**
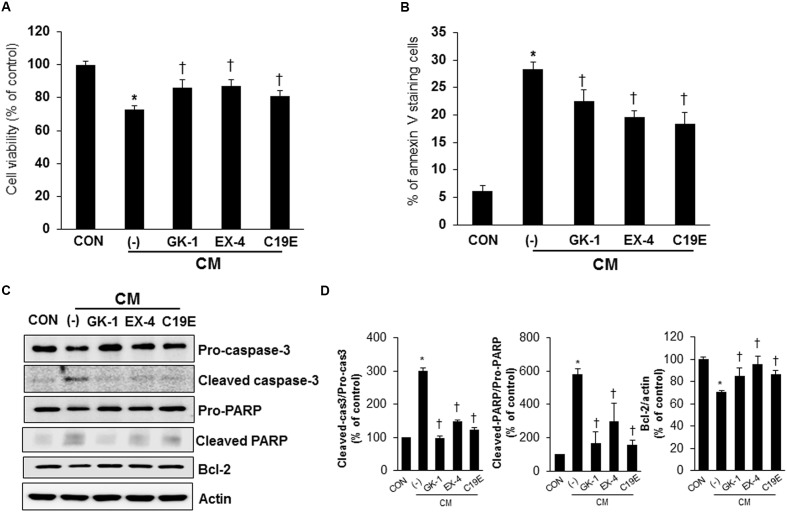
**Anti-apoptotic effect of compound 19e on cytokine-induced toxicity**. INS-1 cells were treated with 20 ng/ml of IL-1β and 20 ng/ml TNF-α (CM) with or without (-) 10 μM of PSN-GK-1 (GK-1), 5 nM of exendin-4 (EX-4) or 5 μM of compound 19e (C19E) for 24 h. **(A)** Cell viability was determined by using a cell counting kit. **(B)** Cells were harvested and stained with annexin-V-phycoerthyrin. Apoptotic cells were counted by flow cytometry. **(C)** Cell lysates were subjected to western blot analysis using specific antibodies. **(D)** Densities of western blot signals were measured using Image J software and relative expression was normalized to actin. The results represent the mean ± SEM from triplicate experiments. ^∗^*P* < 0.05 vs. CON, ^†^*P* < 0.05 vs. CM alone.

As compound 19e treatment reduced cytokine-induced beta-cell death, we investigated whether compound 19e could protect beta-cells against apoptosis induced by the cytokine mixture and the effect was compared to treatment with PSN-GK1 or exendin-4 (Ferdaoussi et al., 2008; Oh et al., 2014). In a preliminary study, we found that 10 μM of PSN-GK1 or 5 nM of exendin-4 effectively suppressed cytokine-induced beta cell apoptosis (Oh et al., 2013). We confirmed that compound 19e (5 μM) decreased cytokine-induced beta-cell death and this effect was similar to that with PSN-GK1 or exendin-4 (**Figure 3A**). The occurrence of apoptosis and the protein levels of apoptosis-related proteins were evaluated in cytokine mixture-treated cells in the absence or presence of compound 19e. The number of annexin V stained cells was significantly increased by cytokine toxicity, but a reduction was observed after treatment with 5 μM of compound 19e (**Figure 3B**). Moreover, the protein levels of cleaved caspase-3 and cleaved PARP induced by the cytokine mixture was significantly reduced by compound 19e treatment (**Figures 3C,D**). The anti-apoptotic protein, Bcl-2, was increased in cytokine-treated cells subjected to compound 19e treatment. Similar results on apoptosis and expression of proteins were seen with PSN-GK1 and exendin-4 (**Figures 3B–D**).

### Compound 19e Prevented Cytokine-Induced Apoptosis by Recovery of Mitochondrial Dysfunction

**FIGURE 4:**
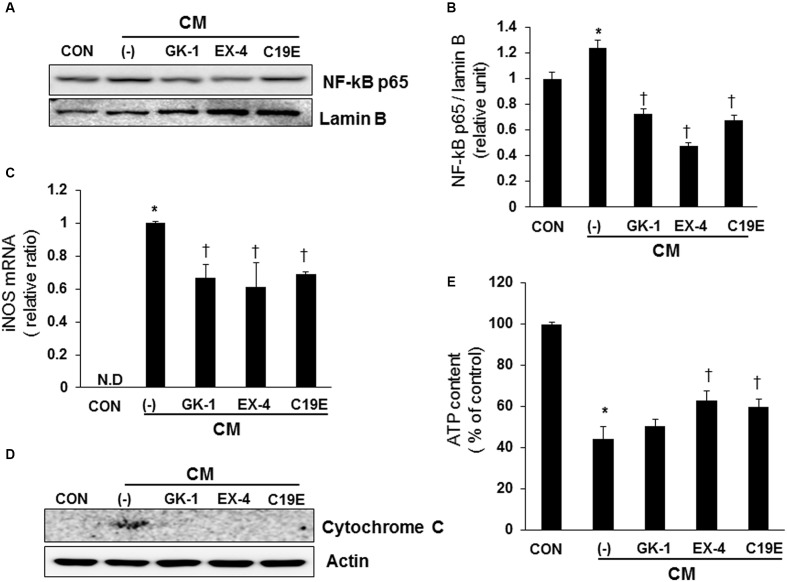
**The protective effect of compound 19e against cytokine-induced mitochondrial dysfunction by suppression of NF-κB signaling**. INS-1 cells were treated as described in **Figure 2**. **(A)** Cell lysates were fractionated and the nuclear fraction was subjected to western blot analysis using specific antibodies. **(B)** Densities of western blot signals were measured using Image J software and relative expression was normalized to lamin B. **(C)** iNOS mRNA levels were analyzed by qRT-PCR and cyclophilin was used as a loading control. N.D, non-detected. **(D)** Cell lysates were subjected to western blot analysis using specific antibodies. Actin was used as a loading control. **(E)** Intracellular concentrations of ATP were determined using an ATP-dependent luminescent cell viability assay. Mean ± SEM from triplicate experiments. ^∗^*P* < 0.05 vs. CON, ^†^*P* < 0.05 vs. CM alone.

It has been reported that cytokines stimulate NF-κB-mediated NO production (Eizirik et al., 1996; Darville and Eizirik, 2001), and the upregulation of NO causes mitochondrial dysfunction in beta-cells (Cetkovic-Cvrlje and Eizirik, 1994). Therefore, the effect of compound 19e on cytokine-stimulated NF-κB activation was evaluated. Cells treated with cytokines alone showed increased transcriptional activity of NF-κB p65, as demonstrated by western blot after nuclear fractionation. However, cytokine-treated cells also treated with compound 19e, PSN-GK1, or exendin-4 showed a significant decrease in NF-κB p65 protein levels (**Figures 4A,B**). Similar to the results obtained with NF-κB activity, cytokines alone upregulated iNOS mRNA levels, and compound 19e treatment downregulated this effect (**Figure 4C**). Finally, to determine whether mitochondrial function was correlated with the anti-apoptotic effect of compound 19e, cytochrome c release was measured, which is a primary indicator of mitochondrial function in beta-cells (Ma et al., 2012). As shown in **Figure 4D**, treatment of cells with cytokines alone for 24 h increased the release of cytochrome c into the cytosol, and this release was decreased by co-treatment with compound 19e. Total cellular ATP content was significantly decreased by cytokine toxicity, but was attenuated in cells treated with 5 μM of compound 19e (**Figure 4E**). Exendin-4-treated cells also demonstrated recovery from mitochondrial dysfunction induced by cytokine treatment (**Figure 4E**).

### Compound 19e Reduces Cytokine-Induced Apoptosis via Modulation of SIRT1 Activity

**FIGURE 5:**
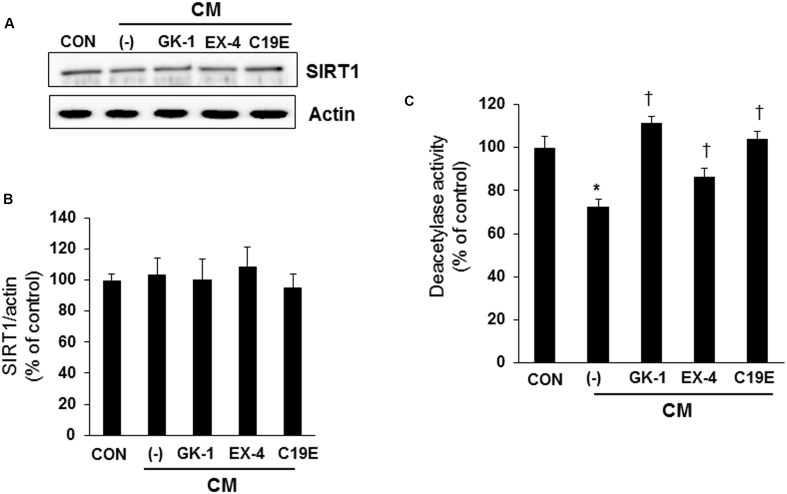
**Effect of compound 19e on SIRT1 deacetylase activity**. INS-1 cells were treated as described in **Figure 2**. **(A)** Cell lysates were subjected to western blot using specific antibodies. **(B)** Densities of western blot signals were measured using Image J software and relative expression was normalized to actin. **(C)** Nuclear protein was extracted and SIRT1 deacetylase activity was measured using fluorogenic substrate. The results represent the mean ± SEM from triplicate experiments. ^∗^*P* < 0.05 vs. CON, ^†^*P* < 0.05 vs. CM alone.

Since SIRT1 has been reported to interfere with NF-κB signaling and exhibit anti-inflammatory actions (Yang et al., 2007), we investigated whether SIRT1 is involved in the anti-apoptotic effect of compound 19e. Western blot analysis was performed, and the enzyme activity of cytokine-treated cells co-treated with GKAs or exendin-4 was examined. SIRT1 expression did not differ between the control and cytokine-treated cells (**Figures 5A,B**). However, deacetylase activity was significantly reduced in cells treated with cytokines alone, and compound 19e attenuated the reduction of SIRT1 activity (**Figure 5C**).

**FIGURE 6:**
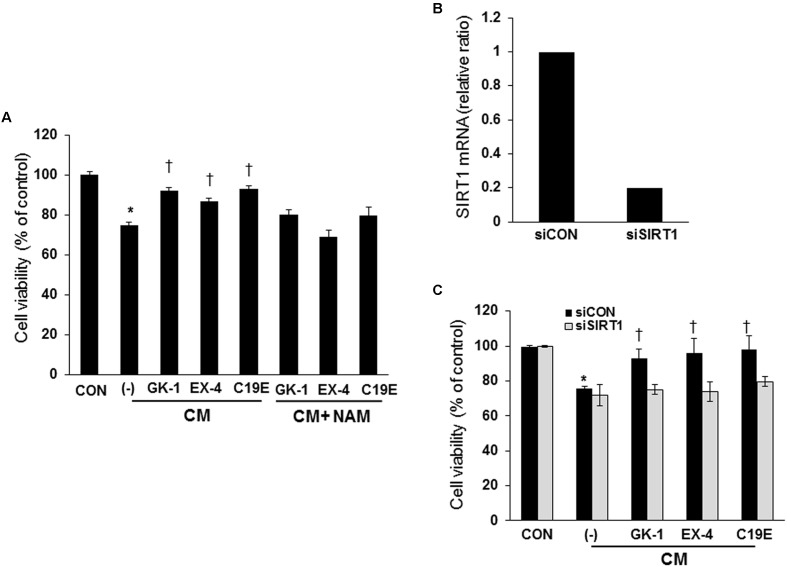
**Amelioration of cytokine-induced cell death by SIRT1 activity inhibitor or SIRT1 siRNA treatment. (A)** INS-1 cells were pre-treated with 2.5 mM nicotinamide (NAM) for 12 h and incubated with GK-1, EX-4, or C19E in the presence of CM for 24 h. Cell viability was determined by using a cell counting kit. **(B)** SIRT1 siRNA (siSIRT1) or scrambled siRNA (siCON) was transfected into INS-1 cells and SIRT1 mRNA levels were analyzed by qRT-PCR. Cyclophilin was used as a loading control. **(C)** Cells were transfected with SIRT1 siRNA (siSIRT1) or scrambled siRNA (siCON) for 24 h and then incubated with GK-1, EX-4, or C19E in the presence of CM for 24 h. Cell viability was determined by using a cell counting kit. Results represent the mean ± SEM from triplicate experiments. ^∗^*P* < 0.05 vs. CON, ^†^*P* < 0.05 vs. CM alone.

To confirm that SIRT1 activation is involved in the anti-apoptotic effect of compound 19e, cells were treated with a SIRT1 deacetylase inhibitor or SIRT1 siRNA, and cell viability was determined. As shown in **Figure 6A**, treatment with the SIRT1 deacetylase inhibitor, nicotinamide (2.5 mM), inhibited the protective effect of compound 19e on cytokine-induced cell death (**Figure 6A**). Similarly, both PSN-GK1 and exendin-4 also significantly activated SIRT1 deacetylase activity, and nicotinamide treatment abolished the protective effect of these compounds (**Figure 6A**). SIRT1 expression was dramatically reduced after 24 h of SIRT1 siRNA transfection (**Figure 6B**), and the protective effect of compound 19e treatment on cytokine-induced cell death was inhibited in SIRT1 siRNA-treated cells compared with scrambled siRNA-treated cells (**Figure 6C**).

## Discussion

Glucokinase is an attractive target for the treatment of type 2 diabetes owing to its anti-apoptotic and proliferative effects on pancreatic beta-cells. A number of allosteric small-molecule GKAs have been developed, and their efficacy has been investigated on beta-cells and in diabetic mice. Previously, compound 19e was discovered as a novel glucokinase, demonstrating hypoglycemic effects in diabetic *db*/*db* mice (Park et al., 2014). In this study, the anti-apoptotic effects and mechanisms of compound 19e were investigated in INS-1 beta-cells treated with cytokines to induce cell death.

It has been reported that GKAs prevent beta-cell death induced by oxidative stress, glucotoxicity, and ER stress. Futamura et al. (2012) demonstrated that Cpd-C (a synthetic GKA compound) increases NADH production and results in prevention of hydrogen peroxide-induced beta-cell apoptosis. GKA 50 and YH-GKA prevent INS-1 cell loss induced by chronic high glucose, and the effects were probably as a result of increase of BAD protein and interaction with glucokinase and voltage-dependent anion channel proteins within the mitochondrial membrane (Wei et al., 2009; Oh et al., 2014). Also, GKA ameliorates ER stress-induced apoptosis by upregulation of IRS-2 expression (Shirakawa et al., 2013). In this study, we demonstrated a previously unidentified function of GKAs – the ability to protect against cytokine-induced beta-cell apoptosis.

One of the important pathogenetic mechanisms of beta-cell damage during diabetes is the increased expression of proinflammatory cytokines such as IL-1β, interferon (IFN)-γ, and TNF-α (Cnop et al., 2005). During the development of type 2 diabetes, there is an increased secretion of TNF-α from infiltrated macrophages in the adipose tissues, which induces the production of several inflammatory cytokines, including IL-1β (Hanafusa and Imagawa, 2008). It has been demonstrated by previous studies and our group that treatment of insulinoma cells or isolated islets with cytokine mixtures reduces cell viability and insulin secretion in response to glucose (Kim et al., 2007; Oh et al., 2011). Therefore, the preventive effect of compound 19e against the deleterious effects of proinflammatory cytokines would be beneficial to preserve functional beta-cell mass. Moreover, compound 19e treatment increased the number of beta-cells via upregulation of IRS-2 expression (data not shown), suggesting that a proliferative effect as well as an anti-apoptotic effect might be mechanisms by which compound 19e induced significant reduction in blood glucose levels in *db/db* mice (Park et al., 2014).

Under diabetic conditions, IL-1β activates NF-κB and consequently regulates the expression of proinflammatory genes, such as Fas, and iNOS (Eizirik et al., 1996; Darville and Eizirik, 2001). Moreover, cytokine-induced NF-κB activation disrupts the mitochondrial membrane potential, which is prevented by overexpression of the anti-apoptotic protein Bcl-2 (Barbu et al., 2002). Therefore, inhibition of NF-κB activation protects pancreatic beta-cells against cytokine-induced apoptosis (Eizirik and Mandrup-Poulsen, 2001; Heimberg et al., 2001). Compound 19e protected INS-1 cells from cytokine toxicity via the suppression of NF-κB-dependent iNOS expression and cytochrome c release, thereby enhancing ATP content.

SIRT1, a class III histone/protein deacetylase, interferes with the NF-κB signaling pathway and thereby exhibits anti-inflammatory and anti-apoptotic properties (Yang et al., 2007). Lee et al. (2009) reported that SIRT1 protein levels were downregulated in RIN (rat insulinoma) cells treated with a cytokine mixture (combination of TNF-α and IFN-γ), and the overexpression of SIRT1 protected against cytokine toxicity by suppressing NF-κB. However, it was reported that SIRT1 deacetylase activity, rather than SIRT1 protein levels, is more relevant in the progression of diabetes and its complications (Nascimento et al., 2013). We observed that SIRT1 activity, but not protein levels, decreased in cytokine-treated cells and compound 19e recovered SIRT1 activity. Moreover, inhibition of SIRT1 activity by nicotinamide or SIRT1 siRNA treatment did not enhance the viability in cells treated with compound 19e. Resveratrol, a SIRT1 activator, exhibits protective actions against cytokine-induced beta-cell dysfunction by activating nicotinamide-dependent protein deacetylase SIRT1 (Lee et al., 2009). These results suggest that compound 19e enhanced SIRT1 activity and consequently inhibited cytokine-induced NF-κB signaling pathways in INS-1 cells.

The mechanism by which compound 19e increases SIRT1 deacetylase activity is unclear, however, a study showing that SIRT1 activation upregulates glucokinase in beta-cells (Vetterli and Maechler, 2011) suggests that GKA is associated with SIRT1 activity. SIRT1 is not only regulated by the NAD^+^/NADH ratio, but also by endogenous proteins involved in signal transduction (Nogueiras et al., 2012). It will be investigated whether compound 19e directly or indirectly regulates SIRT1 activity.

A previous study has shown that exendin-4 attenuates beta-cell apoptosis via the phosphoinositide 3-kinase/protein kinase B (PI3K/PKB), cyclic adenosine monophosphate – protein kinase A (cAMP-PKA), c-jun-N-terminal kinase (JNK), and p38-MAPK pathways (Lee and Jun, 2014). Especially, under conditions of cytokine-induced (IL-1β and/or TNF-α) apoptotic cell death, NF-κB and PKA-dependent JNK phosphorylation are involved in the protective activity of exendin-4 in a mouse beta-cell line, MIN-6 cells (Natalicchio et al., 2010; Liu et al., 2013). The anti-apoptotic effect of PSN-GK1 is not well-known; however, GKAs possibly prevent beta-cell death via regulation of IRS-2 (Shirakawa et al., 2013). In this study, we found that SIRT1-mediated inhibition of NF-κB was involved in the protective effect of exendin-4 and PSN-GK1 against cytokine toxicity. Therefore, it would be interesting to investigate whether a combined treatment of exendin-4 and GKAs exerts additive effects to reduce glucose levels and beta-cell apoptosis in diabetic mice.

## Conclusion

Compound 19e inhibited apoptosis induced by a cytokine mixture in INS-1 cells, and SIRT-1 mediated downregulation of NF-κB and iNOS was involved in this effect. These results suggest that compound 19e may be a potential therapeutic drug to preserve beta-cell mass, at least in part, for the treatment of type 2 diabetes.

## Author Contributions

YO and H-SJ conceived and designed the experiments; YO, ES, and KP performed the experiments; YO, ES, KP, and H-SJ participated in data interpretation and wrote the manuscript; H-SJ contributed reagents/materials/analysis tools and revised the paper.

## Conflict of Interest Statement

The authors declare that the research was conducted in the absence of any commercial or financial relationships that could be construed as a potential conflict of interest.
